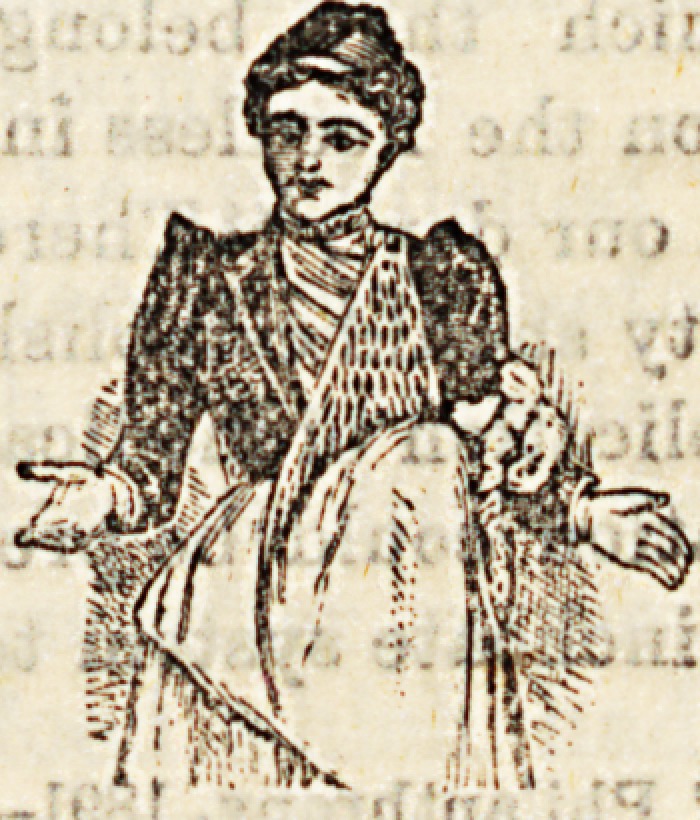# New Drugs, Appliances, and Things Medical

**Published:** 1891-10-24

**Authors:** 


### NEW DRUGS, APPLIANCES, AND THINGS MEDICAL.

[All preparations, appliances, novelties, etc., of which a notice is desired, should be sent for The Editor, to care of The Manager, 140, Strand, London, W.C.]

### THE BABY CARRIER.

The accompanying illustration shows a novelty in hammocks. It is surprising that perambulators and every kind of wheeled vehicle for the transport of babies should have been discovered so many years ago, whilst nobody thought of suspending a net from the shoulders of mother or nurse for the comfort of the baby. Not comfort only, but safety is assured by the baby carrier. And what a relief to the weary mother of many children who has her own housework to do and no nurse. What a comfort, too, to the nurse who has to carry the baby for the first three or four months before it is old enough for the perambulator. The baby carrier only costs 2s. 6d., a first-rate recommendation. Certainly it is one of those indispensables which "no family should be without." It may be obtained of Messrs. Matthews, of John Bright Street, Birmingham.

## Figures and Tables

**Figure f1:**